# Dr. Cappe, in Reply to Mr. Franks

**Published:** 1801-01

**Authors:** Robert Cappe

**Affiliations:** York


### *To the Editors of the Medical and Phyfical Journal.*

Gentlemen,

In the following obfervations on a letter from Mr. Franks, inferted in the laft Number of your Journal, I hope I do not improperly or unhandfomely anticipate his promifed communication.

As Mr. Franks fays that, in fpeaking of the inoculated Cow-pox and Small-pox, I have *magnified the character of one at the expence of the other*, I have carefully read again what I have written, and am, in confcience, obliged fairly to acquit myfelf of this charge. But I will not affume the right of a jury; permit me to lay the facts before your readers, not indeed to juftify myfelf, for it is probable none will care whether I am right or wrong, but becaufe Mr. Franks, without ftating the premifes, has hurried to a conclufion, contradictory to the general belief of the whole medical profeffion, and oppofed to the opinion of fome eminent members of that body, who, through

---

through a feries of years, have made the contagion of Small-pox the fubject of continued obfervation and enquiry.

Mr. Franks admits that the Vaccine Inoculation introduces a difeafe milder than the inoculated Small-pox; and he admits that the Cow-pox are not contagious: In this we are agreed. Mr. Franks himfelf acquits me of having exaggerated the merits of the Cow-pox; and moft of your readers, I think, will acquit me of the charge of detracting from the character of the inoculated Small-pox; for, in the paragraph quoted by Mr. Franks, I have expreffed myfelf much lefs ftrongly than Dr. Haygarth in his Sketch of a Plan to exterminate the Small-Pox; and his opinion cannot be fuppofed to turn to any unfavourable bias, fince his purpofe was to recommend inoculation to check the fpreading of contagion. Having lately read his works with uncommon intereft, I have unconfcioufly fallen into expreffions not much unlike his; the following are his words: "As far as my circle of obfervation extends, both in England and Wales, this improved method of communicating the diftemper has manifeftly appeared to be injurious to the poor, though eminently ufeful to the rich. It has become prejudical to the community, though human art never beftowed fo valuable a bleffing as it confers on the few intelligent individuals who adopt it. It has been falfely attributed by inattentive obfervers, to the inoculated patients fpreading the cafual infection, an event which, in a few inftances, might inadvertently happen." In thefe words Dr. Haygarth acknowledges the fact implied in my letter; and alfo, his belief, that the inoculated Small-pox are infectious. In other parts of his works he has fhewn, that the cafual Small-pox owe their prevalence lefs to effluvia arifing immediately from the patient, than to contagious matter adhering to clothes and other articles in common ufe. Now, it is well known, that the matter contained in the puftules that arife in the inoculated Small-pox does, in no refpect, differ from the matter generated in the cafual difeafe; it muft, therefore, in proportion to the quantity, be equally liable to be diftributed. In general, the puftules are not fo numerous, and the quantity of infectious matter generated, is not fo great in the inoculated as in the cafual Small-pox: the chance, therefore, of the difeafe being conveyed from one perfon to another, is lefs; but it does not vanifh into actual fecurity. Yet Mr. Franks, without referve and without proof, affirms it to be "a common prejudice, that the variolous inoculation gives origin to a contagious difeafe." And he denies that the Cow-pox poffefs any advantage over the Small-pox in this refpect. This opinion not only contradicts general belief, and fhould therefore not be be lightly received; but it cannot be eftablifhed by any number of inftances in which the inoculated Small-pox have not fpread infection, fo long as any inftance appears, in which contagion can be traced to fuch fource. In examining this queftion it fhould not be forgotten that Dr. Haygarth has adduced decifive proof, that the contagion of the moft malignant cafual Small-pox may be circumfcribed, and prevented from fpreading even to the neareft neighbour.

If any perfon doubt whether fociety may derive advantage from the introduction of the Cow-pox, the doubt fhould be openly and fairly ftated, with the facts on which it is founded. If it be ill-founded, it cannot be too quickly difpelled; if it be well-founded, it cannot be too foon or too generally known. But I muft be excufed in thinking it unjuftifiable to publifh, without proof, opinions that may lead any one to difregard or undervalue the advantages that many cautious and experienced perfons expect from the introduction of the Cow-pox.

Mr. Franks is perhaps, in general, right in the opinion, that the air is not contaminated to an injurious degree by inoculation; that probably happens only, when a confluent eruption or numerous puftules are produced. Yet there are many other ways in which contagion may be conveyed from the fick to thofe who have not had the difeafe. Thefe facts fuggefted, and I am forry they juftify, the expreffions in the paragraph to which Mr. Franks objects.

If Mr. Franks's opinion be erroneous, he leads us to imagine our friends fecure while furrounded by danger. A vigilant caution fhould ever prefide over fpeculations in affairs that nearly concern the lives of our fellow-creatures. I am,

GENTLEMEN,

With great refpect,

